# Multigene disruption in undomesticated *Bacillus subtilis* ATCC 6051a using the CRISPR/Cas9 system

**DOI:** 10.1038/srep27943

**Published:** 2016-06-16

**Authors:** Kang Zhang, Xuguo Duan, Jing Wu

**Affiliations:** 1 State Key Laboratory of Food Science and Technology, Jiangnan University, 1800 Lihu Avenue, Wuxi, 214122, China; 2 School of Biotechnology and Key Laboratory of Industrial Biotechnology Ministry of Education, Jiangnan University, 1800 Lihu Avenue, Wuxi, 214122, China

## Abstract

*Bacillus subtilis* ATCC 6051a is an undomesticated strain used in the industrial production of enzymes. Because it is poorly transformable, genetic manipulation in this strain requires a highly efficient genome editing method. In this study, a *Streptococcus pyogenes* CRISPR/Cas9 system consisting of an all-in-one knockout plasmid containing a target-specific guide RNA, *cas9*, and a homologous repair template was established for highly efficient gene disruption in *B. subtilis* ATCC 6051a. With an efficiency of 33% to 53%, this system was used to disrupt the *srfC*, *spoIIAC*, *nprE*, *aprE* and *amyE* genes of *B. subtilis* ATCC 6051a, which hamper its use in industrial fermentation. Compared with *B. subtilis* ATCC 6051a, the final mutant, BS5 (Δ*srfC*, Δ*spoIIAC*, Δ*nprE*, Δ*aprE*, Δ*amyE*), produces much less foam during fermentation, displays greater resistant to spore formation, and secretes 2.5-fold more β-cyclodextrin glycosyltransferase into the fermentation medium. Thus, the CRISPR/Cas9 system proved to be a powerful tool for targeted genome editing in an industrially relevant, poorly transformable strain.

*Bacillus subtilis*, a well-characterized gram-positive bacterium, has been widely used for the production of heterologous proteins. This species and some of its close relatives have excellent protein secretory capability and are generally recognized as safe (GRAS), making them important hosts for the production of antibiotics, medicinal proteins, and industrial enzymes. *B. subtilis* 168 is a model laboratory strain that carries many mutations that have occurred during its modification via irradiation and selection^1^. These modifications have made the organism tryptophan-deficient and improved its transformablility. The *B. subtilis* strains commonly used for recombinant protein production, such as WB600 and WB800, were constructed on the basis of *B. subtilis* 168^2^.

Because the recombinant protein productivity of *B. subtilis* ATCC 6051a is superior to that of *B. subtilis* 168, it has been widely applied to the production of industrial enzymes^3^,^4^. However, *B. subtilis* ATCC 6051a has some undomesticated properties that hamper the extracellular production of recombinant proteins. In particular, it can produce large amounts of foam, highly resistant spores, multiple types of extracellular protease, and high level of amylase during fermentation, which related to *srfC*^5^, *spoIIAC*^6^, *nprE*^7^, *aprE*^8^ and *amyE*^9^, respectively. To improve the usefulness of this important strain, we sought to modify these properties by inactivating the five genes. *B. subtilis* ATCC 6051a is poorly transformable, compared with laboratory strains, because it harbours an 84-kb endogenous plasmid pBS32, which encodes a single-pass trans-membrane protein ComI that inhibits the competence of DNA uptake^10^. Due to its poor competence, the genetic manipulation of *B. subtilis* ATCC 6051a is difficult and require a highly efficient genome editing method. The genome sequence of *B. subtilis* ATCC 6051a was recently determined by Jeong *et al*.^11^, which facilitates genetic manipulation.

Clustered regularly interspaced short palindromic repeat (CRISPR) systems, which are composed of CRISPR RNAs (crRNA), trans-activating CRISPR RNAs (tracrRNAs) and CRISPR-associated (Cas) proteins constitute an immune system in bacteria and archaea that efficiently cleaves foreign DNA entering the cell, including phages and plasmids^12^. Some CRISPR/Cas systems require multiple proteins^13^, whereas the type II CRISPR/Cas system requires a single nuclease; Cas protein 9 (Cas9). In the widely used *Streptococcus pyogenes* type II CRISPR/Cas system^14^, the 20-bp complementary region (N20) within the crRNA guide Cas9 nuclease to its specific target, a roughly 20-nt sequence known as the protospacer, which contains a specific protospacer-adjacent motif (PAM) at its 3′ end^15^. The PAM sequence leads cas9 to create a double-strand break at protospacer (target) sequence^14^,^16^, and it was repaired through homologous recombination using a repair template that is supplied along with the CRISPR/Cas9 system^17^. Recently, a single chimeric guide RNA (sgRNA) containing features of both crRNA and tracrRNA has been developed^18^, which simplify the genome editing design. As an efficient genome editing technology, the type II CRISPR/Cas9 system has been proved to be feasible in point mutation, single gene deletion/insertion and large-size gene cluster deletion^19^. And until recently, it has been widely applied in various organisms including, but not restricted to, *Escherichia coli*^14^, *Streptococcus pneumonia*^14^, *Saccharomyces cerevisiae*^20^, *Lactobacillus reuteri*^21^, *Bombyx mori*^22^, *Drosophila*^23^, mice^24^ and humans cell lines^17^.

Although there have been several successful implementations of the CRISPR/Cas9 system in microbial systems, there have not been any reports of genome editing in *B. subtilis* using a CRISPR/Cas9 system. This study describes the establishment and optimization of a CRISPR/Cas9 system in *B. subtilis* ATCC 6051a. We used this system to disrupt five genes in the *B. subtilis* ATCC 6051a genome (*srfC*, *spoIIAC*, *nprE*, *aprE* and *amyE*) that hamper its use during industrial fermentation. Compared with *B. subtilis* ATCC 6051a, this mutant strain, named BS5, produces much less foam at the level of control, exhibits great resistance to spore formation, and secretes 2.5 times more β-cyclodextrin glycosyltransferase (β-CGTase) into the fermentation medium. β-CGTase is widely used in the production of β-cyclodextrin and mainly produced from wild strains and *E. coli*^25^, which exist low secretion and food safety problem, respectively. The increase of β-CGTase secretion from *Bacillus subtilis* can largely reduce the cost of β-cyclodextrin production, which shows the high industrial value of BS5.

## Results

### Construction of the CRISPR/Cas9 system all in one plasmid

**Figure 1:**
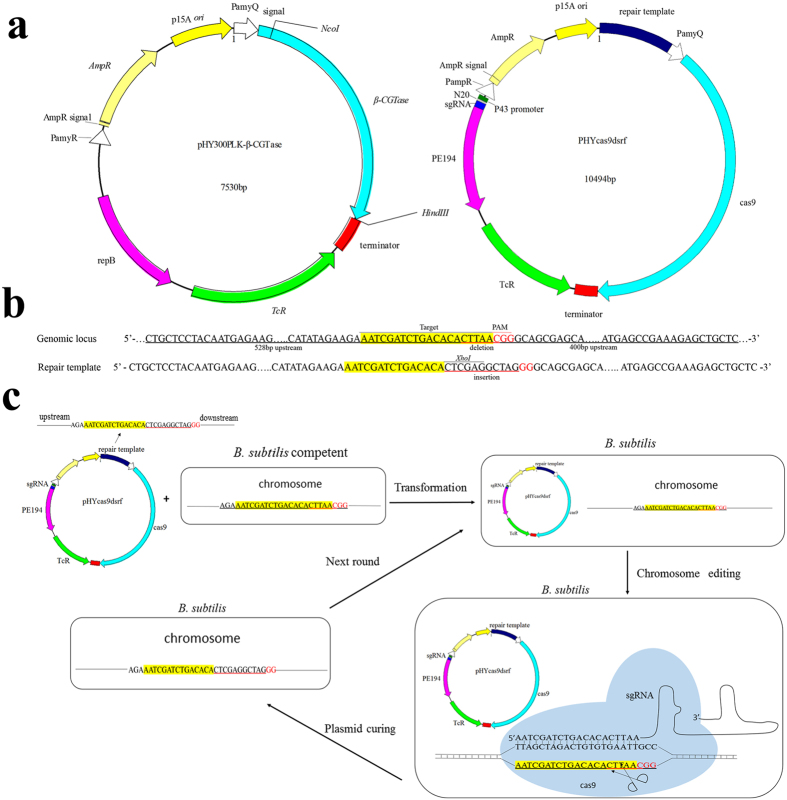
CRISPR/Cas9 system plasmids, homologous repair template and editing procedure. (**a**) Relevant features of pHY300PLK-β-CGTase and the knockout plasmids. p15A *ori*, *E. coli* replication origin; repB, *B. subtilis* replication origin; PE194, *B. subtilis* temperature-sensitive replication origin; *AmpR*, ampicillin-resistance marker; *TcR*, tetracycline resistance marker; PamyR, promoter of ampicillin-resistance marker; *PamyQ*, α-amylase promoter from *B. amyloliquefaciens*; *β-CGTase*, β-CGTase encoding gene; *cas9*, Cas9 encoding gene; sgRNA, target-specific guide RNA; N20, 20-bp complementary region; repair template, homologous repair template obtained by overlap PCR. (**b**) Diagram showing the design of a 934 bp repair template for the specific target within *srfC*. The 6 bp deletion includes 1 bp of the PAM and the last 5 bp of the guide sequence. The 11 bp insertion includes an *Xho* I restriction site and 5 bp of random sequence. (**c**) Detailed diagram of CRISPR/Cas9 system mediated continual genome editing in *B. subtilis* ATCC 6051a.

We assembled a complete CRISPR/Cas9 system in a single knockout plasmid that could be used for highly efficiently genome editing in *B. subtilis* (Fig. 1a). The six knockout plasmids (pHYcas9dsrf1, pHYcas9dsrf2, pHYcas9dspo, pHYcas9dnpr, pHYcas9dapr and pHYcas9damy), which originated from plasmid pHY300PLK-β-CGTase, consist of the *cas9* gene amplified from plasmid pwtcas9-bacterial, a sgRNA and its promoter P43, the temperature-sensitive replicon PE194, and a homologous repair template. The synthesis of Cas9 protein *in vivo* was driven by the α-amylase promoter (*PamyQ*), which originates from *B. amyloliquefaciens*. The P43 promoter is a constitutively expressed promoter^26^ that can drive strong transcription of the sgRNA in *B. subtilis*. The homologous repair template was obtained through overlap extension PCR of regions upstream and downstream of the target locus. The length of the upstream and downstream regions ranged from 450 to 550 bp, and they introduced an *Xho* I site into the target locus. The knockout plasmids and plasmid pHY300PLK-β-CGTase encode ampicillin resistance in *E. coli* and tetracycline resistance in both *E. coli* and *B. subtilis*.

### Disruption of the *srfC* gene using the CRISPR/Cas9 system

**Figure 2:**
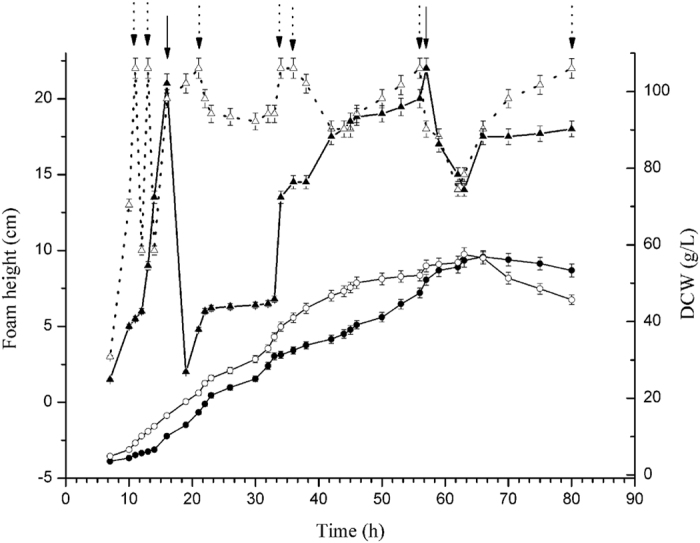
The foam height and cell growth of *B. subtilis* ATCC 6051a and BS1 in 3 L fermenter. During the fermentation, the foam height of *B. subtilis* ATCC 6051a (Δ) and BS1 (▲), and the dry cell weight (DCW) of *B. subtilis* ATCC 6051a (□) and BS1 (■) was measured. The antifoam was added to *B. subtilis* ATCC 6051a (dotted arrow) and BS1 (solid arrow) when foam height reach the limit.

**Figure 3:**
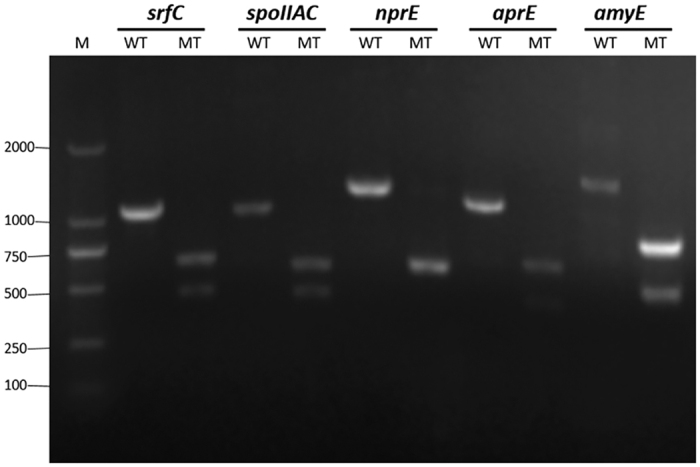
Confirmation of the *srfC* disruption, *spoIIAC* disruption, *nprE* disruption, *aprE* disruption and *amyE* disruption. Digesting the PCR product of upstream and downstream regions with *Xho* I. The digestion products were analysed by agarose gel electrophoresis. Lane M: DNA marker; lane WT: digestion of overlap PCR product using *B. subtilis* ATCC 6051a genomic DNA as the template; lane MT: digestion of overlap PCR product using the indicated gene disruption mutant genomic DNA as the template.

We used *B. subtilis* ATCC 6051a as the initial strain in which to perform genetic manipulations. Fermentation of *B. subtilis* ATCC 6051a in a 3 L fermenter in our laboratory produced a massive amount of foam that requires large quantities of antifoam agent (Fig. 2). Accumulation of the amphiphilic molecule surfactin promotes foam production, and the gene *srfC* is crucial to the regulation of surfactin production^5^. The CRISPR/Cas9 system developed in this study was first tested using *srfC* as the target (Fig. 1c). We used the knockout plasmid pHYcas9dsrf1, which produces both a sgRNA specific to *srfC* and the Cas9 protein, to transform the initial strain, *B. subtilis* ATCC 6051a. Because the temperature-sensitive knockout plasmid is maintained at low copy in transformants at 37 °C, the tetracycline-resistant transformants were confirmed by colony PCR of *cas9*. As for the commonly same genotype of transformation colonies, the target cleavage by Cas9 and homology-directed repair are commonly performed during the liquid incubation of transformation. The resulting mutants had an *Xho* I site within the repair locus, and this disruption genotype passed to the next generation through passaging in liquid media or LB plate. To test the effectiveness of the CRISPR/Cas9 system, the regions upstream and downstream of the repair locus were amplified by PCR, and then the PCR products were digested with *Xho* I (Fig. 3). There are 66 ± 14 transformants after transformation of knockout plasmid pHYcas9dsrf1. 30 transformants were screened, and 13 ± 2 colonies yielded a recombinant genotype with a disruption efficiency of 43% ± 6%. The results, combined with DNA sequencing of the homologous regions of the mutants (Supplementary Fig. S1a) demonstrated that the editing system described above worked efficiently. Curing the knockout plasmid pHYcas9dsrf1 through overnight incubation at 51 °C produced the desired mutant, named BS1. Colonies of BS1 were used as the initial strain for next gene disruption. There are 20 ± 4 transformants after transformation of initial knockout plasmid pHYcas9d, and no *srfC* gene mutant was found among the transformants. Though DNA nonhomologous end joining exist in *B. subtilis*^27^, the low survival rate was probably due to the double-strand breaks in chromosome created by cas9 protein. To test foam production by *B. subtilis* ATCC 6051a and mutant BS1, cells were grown in a 3 L fermenter for 80 h, and the growth rate of *B. subtilis* ATCC 6051a and BS1 has no significant difference. During the whole fermentation process, *B. subtilis* ATCC 6051a produced much foam and required to add antifoam (490 ul) continuously; BS1 produced much less foam at a controllable level that needed 60 ul antifoam. The foam height of *B. subtilis* ATCC 6051a and BS1 was similar after 42 h, while the foam of BS1 shows great sensitive to antifoam (Fig. 2).

In addition to disruption described above, which deleted a relatively small section of the genome, we also designed and attempted a larger deletion of the *srfC* gene. An 1100 bp homologous repair template was designed to have a 500 bp upstream region and 600 bp downstream region flanking the Cas9 cleavage site. Using knockout plasmid pHYcas9dsrf2 that containing this repair template to delete a 284 bp region, which includes 44 bp upstream and 240 bp downstream of the PAM sequence (Supplementary Fig. S2). The efficiency of the 284 bp deletion is 9.1% in *B. subtilis* ATCC 6051a, which is lower than the efficiency of disruption gene. The low efficiency may related to the short length of homologous repair template that result in low homologous recombination efficiency^28^.

### Disruption of *spoIIAC* gene using the CRISPR/Cas9 system

In order to cope with limiting nutrient sources and high cell density, *B. subtilis* can form highly resistant spores^29^ that will germinate and grow in favourable living conditions. These spores drastically hamper *B. subtilis* fermentations and limit their application in the food industry^30^. The *SpoIIAC* gene encodes sigma factor F, which permits cells to proceed through stage II of sporulation^6^,^31^,^32^,^33^. Using knockout plasmid pHYcas9dspo, we disrupted *spoIIAC* gene using the method described above. The resulting mutant, named BS2, was screened by amplifying the upstream and downstream regions and digesting the PCR products with *Xho* I (Fig. 3). The homologous regions of mutant BS2 were also subjected to DNA sequencing (Supplementary Fig. S1b) to confirm the result. The disruption efficiency of *SpoIIAC* gene was 36% ± 3%. After being cultured at 40 °C for 48 h in a culture medium that favours spore formation, the sporulation efficiency of BS1 was 28.44% (262 colonies/921 colonies), while the sporulation efficiency of BS2 was 0% (0 colonies/182 colonies), which shows that mutant BS2 has great resistant to spore formation.

### Disruption of the *nprE* and *aprE* genes using the CRISPR/Cas9 system

**Figure 4:**
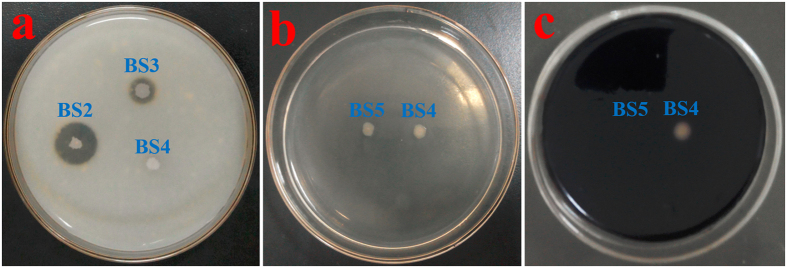
Detection of protease activity and α-amylase activity. (**a**) Transparent rings formed on 5% non-fat powdered milk medium to detect the protease activity of mutants BS2, BS3 and BS4; (**b**) The mutants BS4 and BS5 were grown on LB plate containing 1% soluble starch, then the colonies were wiped; and (**c**) the plate was stained with iodine.

Strains that lack several extracellular protease genes generally show superior extracellular protein productivity^7^,^34^. The *nprE* and *aprE* genes encode alkaline and neutral extracellular proteases, respectively, in *B. subtilis*. We constructed the knockout plasmids pHYcas9dnpr and pHYcas9dapr and used them to sequentially disrupt *nprE* and *aprE* gene of mutant BS2 using the CRISPR/Cas9 method described above. Amplification of the upstream and downstream regions, and then digesting the PCR products with *Xho* I allowed us to select the appropriate mutant, named BS3 with disruption efficiency of 53% ± 6% (Fig. 3). The *nprE* and *aprE* double gene mutant, named BS4 with disruption efficiency of 33% ± 3%. The homologous regions of mutant BS3 and BS4 were subjected to DNA sequencing (Supplementary Fig. S1c,d) to confirm the disruption. When cultured on specify agar plates containing 5% non-fat powdered milk, strain BS3 forms a protein clearance zone smaller than the one formed by its parent, mutant BS2, demonstrating that BS3 has reduced protease activity. And strain BS4 shows substantial inhibition of protein degradation on a 5% non-fat powdered milk plate (Fig. 4a).

### Disruption of the *amyE* gene using the CRISPR/Cas9 system

*B. subtilis* releases many extracellular enzymes during the post-exponential growth phase, and α-amylase is one of the major proteins released^9^,^35^. These proteins hamper the purification of industrial products. We constructed the knockout plasmid pHYcas9damy and used it to disrupt the *amyE* gene of mutant BS4 as described above. After amplifying the sequences upstream and downstream of the target region, the PCR products were digested with *Xho* I to select the *amyE* deletion mutant, which was named BS5 with disruption of 53% ± 6% (Fig. 3). The homologous regions of mutant BS5 were also subjected to DNA sequencing (Supplementary Fig. S1d) to confirm the disruption. The results of a starch-plate assay demonstrate that BS5 fails to release α-amylase activity (Fig. 4b,c).

### Extracellular expression of β-CGTase using *B. subtilis* ATCC 6051a and mutant BS5

**Figure 5:**
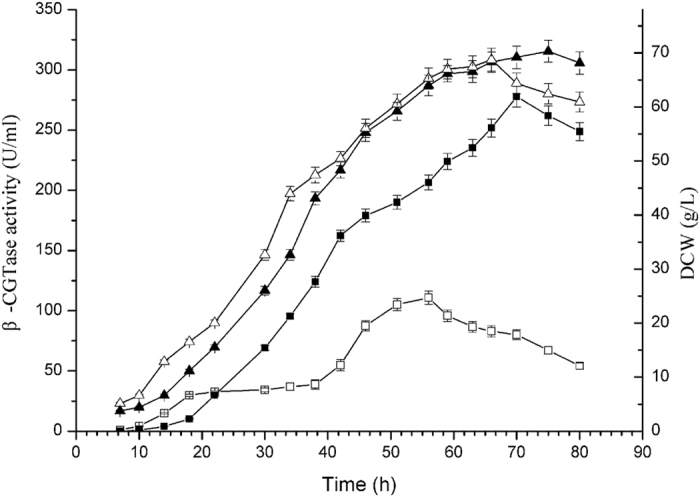
Enzyme assay of β-CGTase expression in *B. subtilis* ATCC 6051a and BS5. During the fermentation, β-CGTase activity of *B. subtilis* ATCC 6051a (□) and BS5 (■), and DCW of *B. subtilis* ATCC 6051a (Δ) and BS5 (▲) was measured.

To evaluate the utility of the strain BS5, in which five genes (*srfC*, *spoIIAC*, *nprE*, *aprE* and *amyE*) have been disrupted, as a host for extracellular recombinant protein expression, the ability of mutant BS5 to produce β-CGTase was compared with that of *B. subtilis* ATCC 6051a. Both expression plasmid pHY300PLK-β-CGTase and empty expression plasmid pHY300PLK were transferred into *B. subtilis* ATCC 6051a and mutant BS5, and the resulting strains were used in an expression study. After 48 h of cultivation in TB medium, the culture supernatants of *B. subtilis* ATCC 6051a and BS5 that harbouring empty expression vector PHY300PLK show no β-CGTase activity. After 80 h of cultivation in 3 L fermenter, the highest β-CGTase activity of *B. subtilis* ATCC 6051a that harbours expression plasmid pHY300PLK-β-CGTase was 110.8 U/ml in 56 h, and the highest dry cell weight (DCW) was 68.8 g/L in 66 h; the highest β-CGTase activity of BS5 that harbours expression plasmid pHY300PLK-β-CGTase was 277.8 U/ml in 70 h, which was 2.5 times of *B. subtilis* ATCC 6051a, and the highest DCW was 70.3 g/L in 75 h (Fig. 5).

## Discussion

In this study, we established a CRISPR/Cas9 system that can disrupt target genes in *B. subtilis* ATCC 6051a (Fig. 1c), and then used this system to construct a mutant strain with improved fermentation characteristics. All of the elements required by the CRISPR system are present in a single plasmid that contains a constitutively expressed *cas9*, a strongly transcribed sgRNA, and a homologous repair template. The sgRNA recognizes a specific site on the *B. subtilis* genome (Supplementary Table S1) and guides the Cas9 protein to the target genome locus, where it creates a double-stranded break. This is followed by homology-directed repair that utilizes a homologous repair template provided by the knockout plasmid. Since the knockout plasmid contains the PE194 temperature-sensitive replicon, it can be easily cured after the mutation step by incubating the mutants at 51 °C overnight. The mutant colonies cured of the knockout plasmid can be used as the parent strain for additional genetic modification. With an efficiency of 33% to 53%, the operate procedure of CRISPR system was simple and time-saving compared with the currently existing *Bacillus subtilis* genome editing methods (Table 1).

The transformation efficiency in *B. subtilis* 168 and *B. subtilis* ATCC 6051a are 1553 ± 213 and 60 ± 13 transformants/μg of the knockout plasmid, respectively. Its poor competence makes genetic manipulation of *B. subtilis* ATCC 6051a inconvenient. To increase the transformability of *B. subtilis* ATCC 6051a, we considered disrupting the gene encoding ComI. However, we were unable to find a target-specific sgRNA target within the 93 bp *comI* gene. The original knockout plasmid (pHYcas9d) did not contain a homologous repair template; therefore, a homologous repair template was transferred into *B. subtilis* ATCC 6051a in the form of PCR fragment, along with the knockout plasmid. This attempt did not meet the efficiency required for genetic editing, perhaps because *B. subtilis* ATCC 6051a was unable to simultaneously take up the knockout plasmid and the homologous repair PCR fragment.

The target recognition of the sgRNA mainly depends on the last 12 bp of the guide sequence; thus, the existence of highly homologous regions in chromosomal DNA may result in off-target effects^36^. These off-target effects have been reported in eukaryotic cells^37^, whereas little attention has been paid to this problem in prokaryotes. Although off-target effects may be less common in bacteria because of their relatively small genome size, bacterial genomes contain some high-homology clusters^38^. There are methods that can be used to reduce the off-target efficiency; for example, designing two sgRNAs to guide the Cas9 protein to cleave the genome at the adjacent sites, using a Cas9 nickase mutant, and making sure the last 12 bp of the guide sequence is highly specific^39^.

Like many *Bacillus* strains, *B. subtilis* produces surfactin, which contains a peptide moiety and a β-hydroxy fatty acid side chain^40^. Because surfactin is an amphiphilic molecule, its accumulation at gas-liquid interfaces can lead to foam production^40^,^41^. The biosynthesis of surfactin is controlled by a non-ribosomal peptide synthase enzyme (SrfC)^42^ and a thioesterase/acyltransferase (SrfD)^43^. The internal thoiesterase domain of srfC, which is a one-module enzyme, controls the conversion of a linear lipoheptapeptide to its cyclic form, as well as the release of surfactin^41^. *B. subtilis* ATCC 6051a produces a large amount of foam during fermentation, which has an extreme affect on fermentation process control and may lead to contamination. Compared with its parent strain, the mutant BS1 produces much less foam and at the level of control. This shows that surfactin may be a major mediator of foam formation, and that there is more work to be done to thoroughly inhibit foam production.

Spore development in *B. subtilis* is governed by multiple RNA polymerase sigma factors^32^. Sigma F, encoded by *SpoIIAC* gene, controls the forespore, sigma E controls the early stage of sporulation, and then sigmas G and K control later stages^32^,^33^. *SpoIIAC* nonsense mutations can block the processing of sigma E precursor protein P31 to sigma E and prevent transcription of *spoIID*^31^. The transcription product, SpoIID, is a membrane-anchored enzyme essential for sporulation^6^,^44^. The observation that mutant BS2 shows great resistant to spore formation demonstrates that inactivation of sigma F can largely block the sporulation of *B. subtilis*.

*Bacillus* species produce many different types of extracellular protease to degrade heterologous extracellular proteins^45^, and many protease-deficient strains display favorable heterologous protein production^7^,^34^. *B. subtilis* WB600, which is deficient in six extracellular proteases due to knockouts of the *nprB*, *nprE*, *aprE*, *mpr*, *bpr* and *epr* genes. This strain displays a level of recombinant protein secretion higher than that of its parent strain^46^. Recently, a *B. amyloliquefaciens* strain lacking six extracellular protease genes was constructed. This strain displays improved production of levan and α-amylase^34^. *B. subtilis* releases multiple extracellular enzymes during the post-exponential period; α-amylase is among the major proteins released^35^. Secretion of a large amount of α-amylase by *B. subtilis* makes the isolation and purification of recombinant proteins difficult on an industrial scale. In addition, the massive expression of endogenous α-amylase increases secretion stress and influences the production of recombinant proteins^47^.

In summary, we established a CRISPR/Cas9 system in the poorly transformable strain *B. subtilis* ATCC 6051a, which is an undomesticated strain with favorable growth characteristics. To improve the usefulness of *B. subtilis* ATCC 6051a as an industrial expression host, we disrupted the *srfC*, *spoIIAC*, *nprE*, *aprE* and *amyE* genes with an efficiency of 33% to 53%. Compared with *B. subtilis* ATCC 6051a, the final mutant (BS5) forms less foam during fermentation, displays greater resistant to spore formation, and secretes 2.5 times more β-CGTase. Thus, the CRISPR system developed here can be used to modify industrially relevant strains with high efficiency, and mutant BS5 can be applied as a superior expression host.

## Materials and Methods

### Strains and plasmids

All bacterial strains and plasmids used in this study are described in Table 2. *Escherichia coli* JM109 was used for plasmid construction. Plasmid pHY300PLK-β-CGTase was previously constructed in our laboratory by inserting the β-CGTase gene of *Bacillus circulans* 251 into the *B. subtilis*-*E. coli* shuttle expression vector pHY300PLK (Takara, Dalian, China)^48^. Plasmid pwtcas9-bacterial was purchased from Addgene (Addgene plasmid # 44250)^49^. *B. subtilis* ATCC 6051a was purchased from the American Type Culture Collection (ATCC).

### Reagents and enzymes

PrimeSTAR polymerase, restriction enzymes, calf intestinal alkaline phosphatase, *Dpn* I, T4 DNA ligase, In-Fusion HD Cloning Plus kit and vector (pMD18-T) were purchased from Takara (Dalian, China). The plasmid mini-prep kit, PCR purification kit, and the agarose gel DNA purification kit were purchased from Tiangen Co. Ltd (Beijing, China). DNA sequencing and DNA primer synthesis were performed by Shanghai RuiDi Biological Technology Co. Ltd. (Shanghai, China). Tryptone and yeast extract were obtained from Oxoid (Hampshire, UK). β-cyclodextrin was purchased from Sigma-Aldrich (Milwaukee, WI, USA). Non-fat powdered milk was purchased from BBI Life Sciences (Shanghai, China). Other chemicals were purchased from Sinopharm Chemical Reagent Co. Ltd. (Shanghai, China).

### Media and growth conditions

For routine construction of plasmids and *B. subtilis* mutants, *E. coli*, *B. subtilis* and *B. subtilis* derivatives were cultured in LB medium (10 g/L tryptone, 5 g/L yeast extract and 10 g/L NaCl) at 37 °C with shaking at 200 rpm. To evaluate spore formation, *B. subtilis* mutants BS1 and BS2 were cultured at 40 °C with shaking at 200 rpm for 48 h in a medium containing 30 g/L dextrin, 30 g/L bean peptone, 1 g/L CaCl_2_ and 0.5 g/L NaCl. Ampicillin (100 mg/L) and tetracycline (20 mg/L) were added as needed.

### Fermentation Cultivate condition

Seed culture was obtained by inoculating 100 ul frozen glycerol stock (stored at −80 °C) into 100 ml LB medium and then incubating 12 h at 37 °C with shaking at 200 rpm. As for Shake-flask cultivation, a portion of 5% (v/v) seed culture (2.5 ml) was used to inoculate 50 ml of TB medium (Supplementary material and method), which was then incubated at 30 °C with shaking at 200 rpm for 48 h. While for the fermentation in 3 L fermenter (BioFlo 110, New Brunswick Scientific Co., Edison, NJ) that containing 0.86 L fermentation medium (Supplementary material and method), before a 10% (v/v) seed culture (100 ml) was inoculated, its pH was adjusted to 7.0 with 20% (v/v) H_3_PO_4_ and NH_4_OH, its temperature was adjusted to 37 °C, and it was added with 30 ml 166.7 g/L glucose. After 2 h of inoculation, the inducer of 30 ml 333.3 g/L lactose was added into fermentation culture as needed. The feed solution (Supplementary material and method) was fed at rate 0 to 15 g glucose/h to confirm the glucose concentration was maintained 0.2 to 0.5 g/L. During the fermentation process, the batch cultivation was carried out at 37 °C, pH 7.0, and 30% dissolved oxygen that were maintained by automatically adjusting stirrer speed (300 rpm-900 rpm) and flow rate of air (1.5–4.0 L/min). Tetracycline (20 mg/L) was added as needed every 24 h. The foam height was measured as needed and antifoam was added manually. For both cultivation, the samples collected at certain time intervals were centrifuged at 12,000 × g for 10 min at 4 °C. For DCW determination, the pellet was resuspended with 0.9% (w/v) NaCl and centrifuged at 12000 × g for 10 min, and dried to a constant weight at 105 °C. As for the host strains that harbour expression plasmid pHY300PLK-β-CGTase, the culture supernatant contains β-CGTase.

### Plasmids construction

The sequences of all of the primers used in this study are listed in Table 3. The initial CRISPR system knockout plasmid pHYcas9d was assembled from four fragments (One, Two, Three and Four), each of which overlaps its two adjacent fragments by 15 bp, using the In-Fusion HD Cloning Plus kit^50^. Fragment One, which encodes the Cas9 protein, was amplified from plasmid pwtcas9-bacterial using the primer pair P03/P04. Fragment Two, which includes a P43 promoter for sgRNA expression, a target-specific 20-nt guide sequence specific for *srfC* fused with the sgRNA sequence, and a temperature-sensitive replicon PE194 for plasmid curing, was synthesized and ligated into the cloning vector pMD18-T. This fragment was amplified using the primer pair P07/P08. Fragment Three includes an *E. coli* replication origin (p15A *ori*), an ampicillin-resistance marker (*ampR*) and the α-amylase promoter (*PamyQ*) from *B. amyloliquefaciens*. This fragment was amplified from plasmid pHY300PLK-β-CGTase using primers P02 and P05. Fragment Four includes a tetracycline resistance marker (*TcR*) and a terminator from *B. amyloliquefaciens*. This fragment was amplified from plasmid pHY300PLK-β-CGTase using primers P01and P06. We amplified the upstream and downstream regions of the target locus using *B. subtilis* ATCC 6051A genomic DNA as a template and two primer pairs: P09/P10 and P11/P12. The amplified fragments were ligated together using overlap extension PCR, forming homologous repair template. To form the final knockout plasmid, pHYcas9dsrf1, the appropriate homologous repair template was inserted into the *Xba* I site of pHYcas9d (Fig. 1a). During double-strand repair, this repair template removes 6 bp of native sequence and inserts an *Xho* I restriction site and 5 bp of random sequence (Fig. 1b).

The other five knockout plasmids (pHYcas9dsrf2, pHYcas9dspo, pHYcas9dnpr, pHYcas9dapr and pHYcas9damy) were constructed from pHYcas9dsrf1 by changing the 20-nt guide sequence (except pHYcas9dsrf2) and replacing the homologous repair template. The 20-nt guide sequence was changed using inverse PCR with the four primer pairs P17/P18 (for pHYcas9dspo), P23/P24 (for pHYcas9dnpr), P29/P30 (for pHYcas9dapr), and P35/P36 (for pHYcas9damy), which hanging the new modified 20-nt guide sequence at the 5′ end. The homologous repair templates were created by overlap extension PCR of sequences upstream and downstream of the 20-nt guide sequence. These sequences were amplified from the *B. subtilis* ATCC 6051a genome using primer pairs P13/P14 and P15/P16 (for pHYcas9dsrf2), P19/P20 and P21/P22 (for pHYcas9dspo), P25/P26 and P27/P28 (for pHYcas9dnpr), P31/P32 and P33/P34 (for pHYcas9dapr), and P37/P38 and P39/P40 (for pHYcas9damy). Then the appropriate homologous repair template was inserted into the modified pHYcas9dsrf1 by digesting the PCR product and the modified plasmid with *Xba* I and ligating the appropriate fragments.

### Genome editing

*B. subtilis* competent cells were made by the method of Anagnostopoulos and Spizizen^51^. Tetracycline-resistant transformants were confirmed by colony PCR of the *cas9* gene. Using transformant genomic DNA as the template, verification PCR reactions were carried out using specific primers (Supplementary Table S2) that anneal outside the homologous repair template. PCR products from disruption mutants were identified by digestion with *Xho* I, and the homologous repair regions of the mutants were subsequently verified by DNA sequencing.

### Plasmid curing

To cure the mutants of the knockout plasmid, edited colonies harbouring knockout plasmid were used to inoculate 10 mL of LB medium containing tetracycline (20 mg/L). The culture was incubated at 37  °C, and then streaked onto LB plates that were subsequently incubated overnight at 51  °C^52^. The colonies cured of knockout plasmid were confirmed by streaking them onto LB plates containing tetracycline (20 mg/L); colonies cured of plasmid fail to grow at 37  °C. These colonies were used in the next round of genome editing.

### Calculation of sporulation efficiency

After cultivation in a medium conducive to spore formation at 40  °C for 48 h, Cells of mutants BS1 and BS2 were diluted and divided into two same amount of portions, respectively, one of which was heat at 75  °C for 20 min^53^, then streak them onto LB plates that were subsequently incubated overnight at 37  °C. The cell ratio of two portions was calculated as sporulation efficiency.

### Detection of protease activity

To detect the protease activity of mutants BS2, BS3 and BS4, strains were dropped onto a 5% non-fat powdered milk plate at 37  °C and incubated for 36 h^54^. Under these conditions, secreted proteases form a clear ring around the secreting colony.

### Detection of α-amylase activity

The α-amylase expression of mutants BS4 and BS5 was detected by dropping the strains onto LB plates containing 1% soluble starch at 37  °C for 24 h, then staining the plates with iodine^55^. Under these conditions, secreted α-amylases create a colourless ring around the secreting colony.

### Enzyme assay of β-CGTase

The β-cyclodextrin-forming activity was determined using the observation that β-cyclodextrin forms a stable, colourless inclusion complex with phenolphthalein^56^. A small sample (0.1 ml) of appropriately diluted culture supernatant was incubated with 2 ml of 1% (w/v) soluble starch in 25 mM phosphate buffer (pH 5.5) at 50  °C for 10 min. The amount of β-cyclodextrin formed was determined by titrating the sample with a standard phenolphthalein solution. One unit of activity was defined as the amount of enzyme that produce 1 μmol of β-cyclodextrin per min^57^.

## Additional Information

**How to cite this article**: Zhang, K. *et al*. Multigene disruption in undomesticated *Bacillus subtilis* ATCC 6051a using the CRISPR/Cas9 system. *Sci. Rep.* **6**, 27943; doi: 10.1038/srep27943 (2016).

## Supplementary Material

Supplementary Information

## Figures and Tables

**Table 1:** Time required for *Bacillus subtilis* genome editing by the current existing methods.

Delivery plasmid^58^	Counter-selectable marker^59^	Cre/loxP^28^	CRISPR/Cas9
Plasmid construction (8 days*)	Plasmid construction8 days	Plasmid construction (8 days)	Plasmid construction (9 days)
Transformation (1.5 days)	Transformation (1.5 days)	The PCR fusion of *Spc*/*Zeo* and target homology fragment (1 days)	Transformation (1.5 days)
Marker deletion (3 days)	Counter-selectable marker integration by single cross-over (3 days)	PCR product transformation (1.5 days)	Verification and plasmid curing (2 days)
Verification (1.5 days)	Counter-selectable marker eviction by double cross-over (2 days)	pTSC plasmid transformation and recombination mediated by cre recombinase (1.5 days)	
	Verification (1.5 days)	Verification and plasmid curing (2 days)	
Total 14 days	Total 16 days	Total 14 days	Total 12.5 days

^*^The time in the table was calculated in the least time required for each manipulation.

**Table 2:** Strains and plasmids used in this study.

Strain or plasmid	characteristics	reference
Strains		
* E. coli* JM109	*recA1, endA1, thi, gyrA96, supE44, hsdR17* Δ (*lac-proAB*)/F’[*traD36,proAB*^*+*^*, lacІ*^*q*^*, lacZ*ΔM15]	Takara
*B. subtilis* ATCC 6051a	Wild type	ATCC
BS1	ATCC 6051a derivative, Δ*srfC*	This work
BS2	ATCC 6051a derivative, Δ*srfC*, Δ*spoIIAC*	This work
BS3	ATCC 6051a derivative, Δ*srfC*, Δ*spoIIAC*, Δ*nprE*	This work
BS4	ATCC 6051a derivative, Δ*srfC*, Δ*spoIIAC*, Δ*nprE*, Δ*aprE*	This work
BS5	ATCC 6051a derivative, Δ*srfC*, Δ*spoIIAC*, Δ*nprE*, Δ*aprE*, Δ*amyE*	This work
Plasmids		
pMD18-T	Amp^r^, MCS	Takara
pHY300PLK	Amp^r^ (*E. coli*), Tet^r^ (*B. subtilis* and *E. coli*), *B. subtilis*-*E. coli* shuttle expression vector	Takara
pHY300PLK-β-CGTase	Amp^r^ (*E. coli*), Tet^r^ (*B. subtilis* and *E. coli*), α-amylase promoter (*P* ), β-CGTase gene.	This lab
pwtCas9-bacteria	Amp^r^, tetracycline repressor TetR, *cas9* gene	Stanley Qi
pHYcas9d	Amp^r^ (*E. coli*), Tet^r^ (*B. subtilis* and *E. coli*), *B. subtilis*-*E. coli* shuttle expression vector, PE194 temperature-sensitive replicon, sgRNA of *srfC*	This work
pHYcas9dsrf1	Amp^r^ (*E. coli*), Tet^r^ (*B. subtilis* and *E. coli*), *B. subtilis*-*E. coli* shuttle expression vector, PE194 temperature-sensitive replicon, sgRNA and repair template of *srfC*	This work
pHYcas9dsrf2	pHYcas9dsrf1 derivative with repair template for 284 bp deletion of *srfC*	This work
pHYcas9dspo	pHYcas9dsrf derivative with sgRNA and repair template of *spoIIAC*	This work
pHYcas9dnpr	pHYcas9dsrpf derivative with sgRNA and repair template of *nprE*	This work
pHYcas9dapr	pHYcas9dsrf derivative with sgRNA and repair template of *aprE*	This work
pHYcas9damy	pHYcas9dsrf derivative with sgRNA and repair template of *amyE*	This work

**Table 3:** Primers used in this study.

primers	Sequence (5′−3′)*
P01	TTTCTTATACAAATTATATTTTACATATCAAT
P02	GGCAACCGTAAGCTTGGTAAT
P03	AATTTGTATAAGAAAATGGATAAGAAATACTCAATAGGCT
P04	AAGCTTACGGTTGCCTTAGTCACCTCCTAGCTGACTC
P05	GCGTCTGTACGTTCCTTAAGG
P06	GTAGTTCAACAAACGGGCC
P07	GGAACGTACAGACGCATTTTACATTTTTAGAAATGGGC
P08	CGTTTGTTGAACTACGCAGTCGGCTTAAACCAG
P09	C**TCTAGA**CTGCTCCTACAATGAGAAGGAG
P10	GCTGCCCCTAGCCTCGAGTGTGTCAGATCGATTTCTTC
P11	CTCGAGGCTAGGGGCAGCGAGCAAACAGC
P12	C**TCTAGA**GAGCAGCTCTTTCGGCTCATAG
P13	C**TCTAGA**TAAGCTTCAGCGGCATGAT
P14	CTTTTGTTCGCGCAGAGTCGTGAATGAACACGGTACG
P15	CCGTGTTCATTCACGACTCTGCGCGAACAAAAGCC
P16	C**TCTAGA**ACTGCTTTCTTCATTTTTCGC
P17	TTGTTTGGTCTGTCGTACAGGTTTTAGAGCTAGAAATAGCAAGTTAA
P18	CTGTACGACAGACCAAACAATTATATTTTACATAATCGCGCGC
P19	C**TCTAGA**CGTGTGACAGTTGCTTCATT
P20	AAAAACCCTGGCCTCGAGCGACAGACCAAACAAGACGC
P21	CTCGAGGCCAGGGTTTTTAAACAGAGGATATGAGC
P22	C**TCTAGA**GTCAGCGATTTGATCAAGCA
P23	GCACTCGCTTTCAAAGCTATGTTTTAGAGCTAGAAATAGCAAGTTAA
P24	ATAGCTTTGAAAGCGAGTGCTTATATTTTACATAATCGCGCGC
P25	C**TCTAGA**GTCTGTTGCTGTCGCTGCTT
P26	ATTTGCCTCGATCTCGAGTTTGAAAGCGAGTGCCAGC
P27	CTCGAGATCGAGGCAAATCACCAGACGCTGT
P28	C**TCTAGA**CCGTAAATCATCTGGTCTCC
P29	CCTGACTTAAACGTCAGAGGGTTTTAGAGCTAGAAATAGCAAGTTAA
P30	CCTCTGACGTTTAAGTCAGGTTATATTTTACATAATCGCGCGC
P31	C**TCTAGA**GTGGATCAGCTTGTTGTTTGC
P32	TTGCTCCGGCGTCTCGAGGACGTTTAAGTCAGGATGAGAAG
P33	CTCGAGACGCCGGAGCAAGCTTCGTACCTTC
P34	C**TCTAGA**CCGTAAGTGCCTCCAGGAAG
P35	GTCACGCAGAATTCATTGCTGTTTTAGAGCTAGAAATAGCAAGTTAA
P36	AGCAATGAATTCTGCGTGACTTATATTTTACATAATCGCGCGC
P37	C**TCTAGA**CCTCTTTACTGCCGTTATTCG
P38	ACAGCCCGGAACCTCGAGTGAATTCTGCGTGACATCCC
P39	CTCGAGGTTCCGGGCTGTATGACTGGAATACAC
P40	C**TCTAGA**TTCAAATAAAGCACTCCCGC

^*^The restriction enzyme sites are bold and underlined.
