# Supplementary material for: Multigene disruption in undomesticated Bacillus subtilis ATCC 6051a using the CRISPR/Cas9 system
Source: Sci Rep. 2016 Jun 16;6:27943. doi: 10.1038/srep27943 (PMC4910044; doi:10.1038/srep27943)
Supplement: Supplementary Information [file srep27943-s1.pdf]

Supplemental Informention

Multigene disruption in undomesticated *Bacillus subtilis* ATCC 6051a using the CRISPR/Cas9 system

Kang Zhang[1,2 §], Xuguo Duan[1,2 §] & Jing Wu[1,2,*]

[1]State Key Laboratory of Food Science and Technology, Jiangnan University, 1800 Lihu Avenue, Wuxi, 214122, China.

[2]School of Biotechnology and Key Laboratory of Industrial Biotechnology Ministry of Education, Jiangnan University, 1800 Lihu Avenue, Wuxi, 214122, China.

[§]These authors contributed equally to this work.

[*]Correspondence and requests for materials should be addressed to J.W. (email: jingwu@jiangnan.edu.cn).

Supplemental material and method

Fermentation cultivate medium

TB medium for Shake-flask cultivation that contains 12 g/L tryptone, 24 g/L yeast extract, 5 g/L glycerol, 12.54 g/L K_2HPO_4, 2.31 g/L KH_2PO_4; The fermentation medium in 3 L fermentor was modified mineral salt medium [1], which contains 20 g/L yeast extract, 30 g/L corn steep powder, 1 g/L $(NH_4)_2$-H-citrate, 2 g/L Na_2SO_3, 2.68 g/L $(NH_4)_2SO_4$, 14.6 g/L K_2HPO_4, 4 g/L $NaH_2PO_4 \cdot H_2O$, 1 g/L $MgSO_4 \cdot 7H_2O$, 3 ml/L trace element solution (TES) [2]. The feed solution contained: 500 g/L glucose, 7.89 g/L $MgSO_4 \cdot 7H_2O$, 63.36 g/L $(NH_4)_2HPO_4$ and 40 ml/L TES.

Statistical analysis

All the experiments were performed in triplicate, and data were expressed as the mean ± standard deviation. Using student's test to perform statistical analysis and statistical significance was confirmed at $P < 0.01$ or 0.05.

References

1 Wilms, B. *et al.* High-cell-density fermentation for production of L-N-carbamoylase using an expression system based on the *Escherichia coli* rhaBAD promoter. *Biotechnol Bioeng* **73,** 95-103 (2001).

2 Wenzel, M., Mueller, A., Siemann-Herzberg, M. & Altenbuchner, J. Self-inducible *Bacillus subtilis* expression system for reliable and inexpensive protein production by high-cell-density fermentation. *Appl Environ Micro* **77,** 6419-6425 (2011).

Supplemental figures

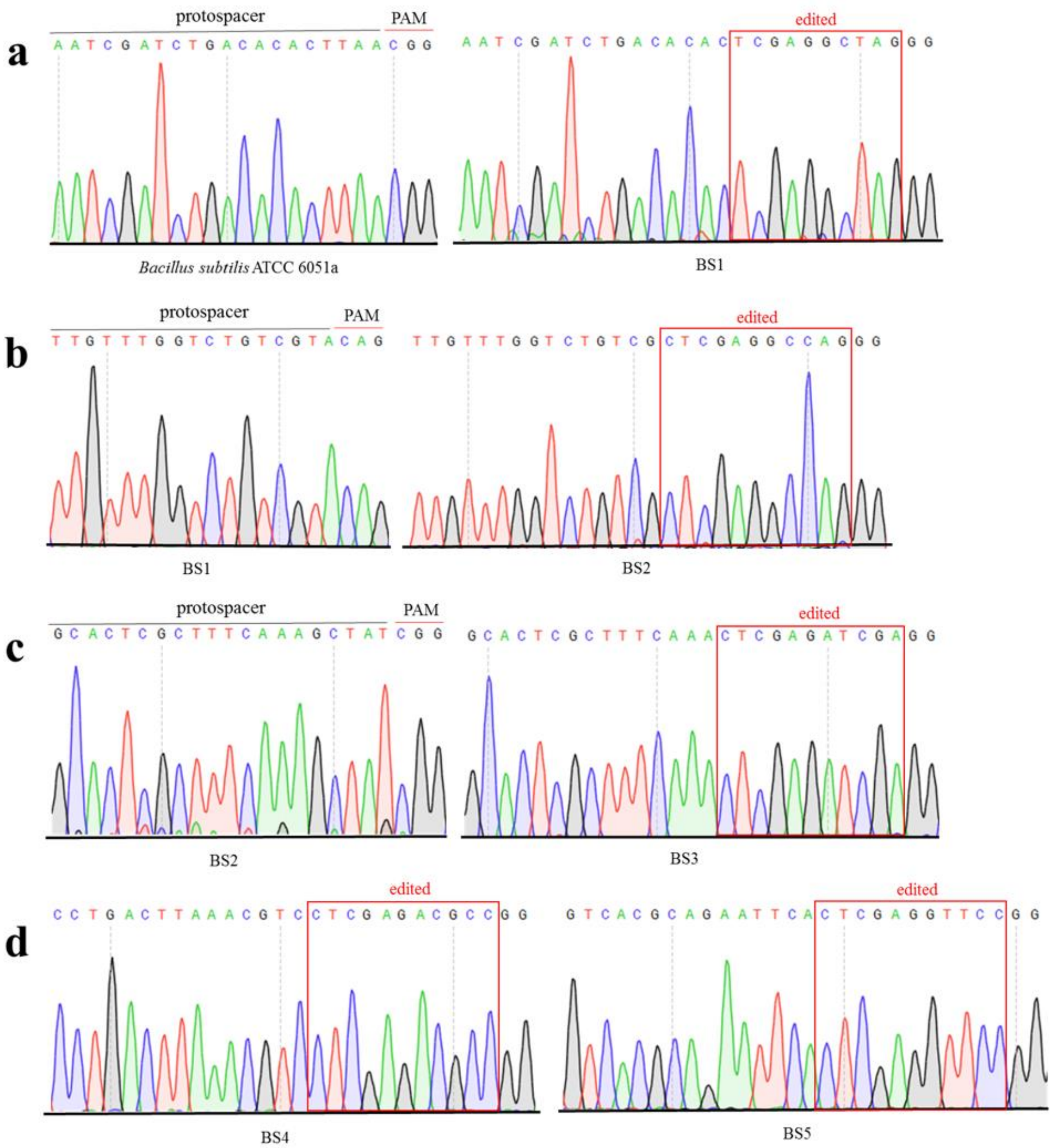

Figure S1. The DNA sequence chromatogram of the homologous regions of *Bacillus subtilis* ATCC 6051a and mutants. (**a**) The DNA sequencing of sgRNA target in *B. subtilis* ATCC 6051a genome and *srfC* gene disruption editing in BS1 genome. (**b**) The DNA sequencing of sgRNA target in BS1 genome and the *spoIIAC* gene disruption editing in BS2 genome. (**c**)The DNA sequencing of sgRNA target in BS2 genome and the *nprE* gene disruption editing in BS3 genome. (**d**) The *aprE* gene disruption editing in BS4 genome and *amyE* gene disruption editing in BS5 genome.

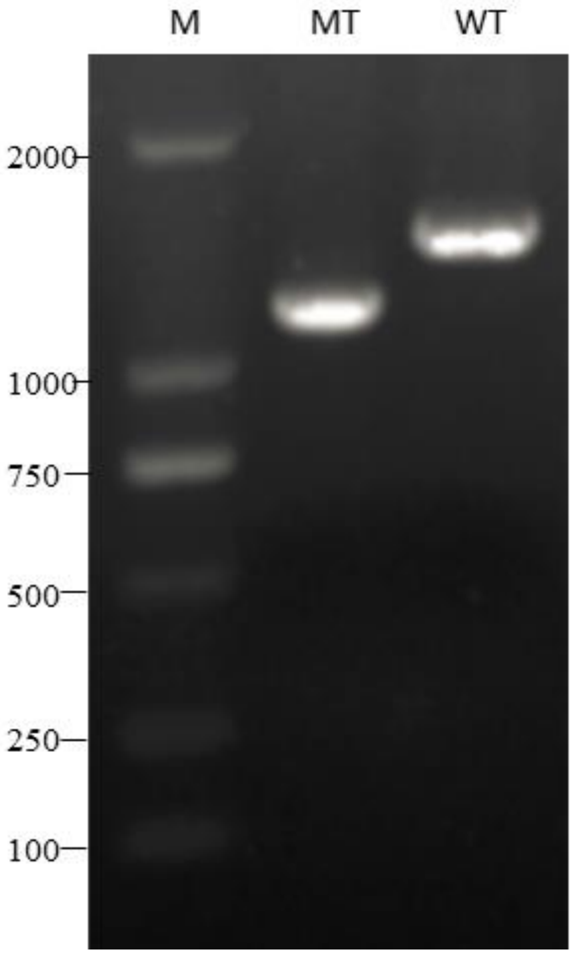

Figure S2. Confirmation of the 284 bp deletion in *srfC* gene. The regions upstream and downstream of the repair locus were amplified by PCR and analysed by agarose gel electrophoresis. Lane M: DNA marker; lane WT: PCR product using *B. subtilis* ATCC 6051a genomic DNA as the template; lane MT: PCR product using the 284 bp deletion mutant strain genomic DNA as the template.

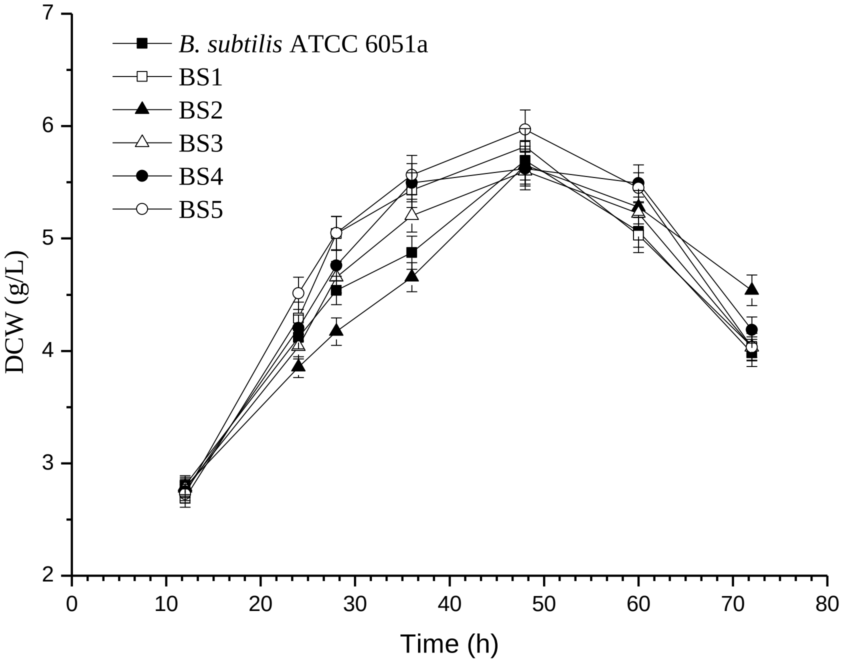

Figure S3. Cell growth of *B. subtilis* ATCC 6051a, BS1, BS2, BS3, BS4 and BS5. During the Shake-flask cultivation in TB medium at 30 ℃, DCW of fermentation culture was measured to evaluate the growth rate.

Supplemental tables

Table S1. The sgRNA sequences of five disruption genes.

Target gene	Sequence (5’-3’)
srfC	AATCGATCTGACACACTTAA
spoIIAC	TTGTTTGGTCTGTCGTACAG
nprE	GCACTCGCTTTCAAAGCTAT
aprE	CCTGACTTAAACGTCAGAGG
amyE	GTCACGCAGAATTCATTGCT

Table S2. Primers used to confirm the disruption or deletion result.

Gene	Forward sequence (5’-3’)	Reverse sequence (5’-3’)
srfC (disruption)	GAACCGTCCGCTTTCGATATG	TTGCAAGCTCGGTAAAGGCC
srfC (deletion)	GAACCGTCCGCTTTCGATATG	AACATCCACCATATCAAAGCCG

spoIIAC	GCACCTTGAGTTTTCTGCCC	TCTTTCAGCGCAATTTTGTC
nprE	CCACCACATGACACTTGACTC	GAGAAGAATGAACCGTCGCC
aprE	GGAGAGGGTAAAGAGTGAGAAG	TTCCAAGATATGTTGCAGTGC
amyE	TGTTTGCAAAACGATTCAAAA	GCATTGATCGTGCCTGTCAG

Supplemental sequences

Gene sequence of sgRNA chimera

GTTTTAGAGCTAGAAATAGCAAGTTAAAATAAGGCTAGTCCGTTATCAACTT
GAAAAAGTGGCACCGAGTCGGTGCTTTTTTTG

Gene sequence of promoter P43

ATTTTACATTTTTAGAAATGGGCGTGAAAAAAAGCGCGCGATTATGTAAAAT
ATAA
